# Atypical Normokalemic Case of Thyrotoxic Periodic Paralysis in a Pediatric Patient

**DOI:** 10.7759/cureus.57467

**Published:** 2024-04-02

**Authors:** Fahad Gadi, Tim Schueler

**Affiliations:** 1 Pediatrics, Faculty of Medicine in Rabigh, King Abdulaziz University, Jeddah, SAU; 2 Pediatrics, Charité – Universitätsmedizin Berlin, Berlin, DEU

**Keywords:** pediatric, normokalemia, grave's disease, periodic paralysis, thyrotoxicosis

## Abstract

Thyrotoxic periodic paralysis (TPP) is a rare complication of hyperthyroidism characterized by typical muscular symptoms, ranging from paresis to complete paralysis, commonly associated with low potassium blood levels (<3 mmol/l). It is more commonly reported in adult Asian individuals and can lead to life-threatening situations. Therefore, early clinical diagnosis and targeted therapy are of crucial importance.

In this article, we report the case of a 17-year-old adolescent with a Vietnamese background and known Graves’ disease who was admitted with typical TPP-related symptoms but no hypokalemia. After treatment with an antithyroid medication and oral potassium supplementation, no new episode of TPP was observed. Using next-generation sequencing, a genetic analysis of TPP-related ion channel genes (KCNJ2, KCNJ18, KCNE3, SCN4A, and CACNA1S) found no known/likely pathogenic variants or variants of unknown significance. To the best of our knowledge, this is only the second reported case of quite normokalemic TPP in the pediatric population. Prompt diagnosis of TPP is essential to prevent harmful complications. Supplementation with potassium appears to be successful alongside non-selective beta-blockers. Normalization of thyroid function should be pursued to prevent new attacks, which is considered the best preventive measure.

## Introduction

Thyrotoxic periodic paralysis (TPP) is a rare complication of uncontrolled hyperthyroidism often associated with Graves’ disease (GD) [1-3]. TPP is more commonly found in males of Asian descent, typically in their second or third decade of life, but is rarely observed in pediatric patients [1,3-5]. Typically, individuals experiencing TPP exhibit low levels of potassium during attacks (<3 mmol/l) [1-3]. Potential triggers include high-carbohydrate meals, heavy physical activity, infections, and exposure to cold weather [1]. Furthermore, non-familial onset is also common [5,6].

Typical symptoms of TPP include symmetric muscular weakness or paralysis, primarily affecting the proximal lower extremities, which can progress to complete quadriplegia, muscle stiffness, muscle aches, and cramps [1,5]. Episodes often occur in the morning or at night [1] and typically last from hours to a few days, with spontaneous recovery even without therapy [1]. However, respiratory failure due to the involvement of the respiratory muscles and cardiac arrhythmias are possible life-threatening complications [1,7].

Potassium supplementation and non-selective beta-blockers are suggested options for therapy during acute attacks or to prevent further episodes [1]. It is essential to mention that the best treatment for TPP is to prevent this complication by maintaining a well-controlled thyroid function [1,2,8].

Important differential diagnoses of TPP include familial hypokalemic periodic paralysis (FHPP), renal/gastrointestinal potassium loss, Guillain-Barré syndrome, transverse myelitis, spinal cord compression, or psychosomatic disorders [4-6,9]. Suitable diagnostics for excluding these diseases are essential, such as blood gas analysis, thyroid function tests, neurological examinations, and body imaging [4-6].

As TPP emerges from hyperthyroidism, it is suggested that due to high levels of thyroid hormones and catecholamines, the Na/K pump becomes hyperactive, resulting in an influx of extracellular potassium and a subsequent decrease in intravascular potassium levels [3]. This immediate potassium shift leads to a loss of membrane potential, resulting in muscular paralysis [10]. However, according to several case reports, total body potassium levels do not seem to be reduced in every case [1,3-7,11,12].

Gene alterations in genomic regions of ion channels cohesive to the Na/K pump, such as KCNJ18, KCNJ2, SCNA4, CACNA1S, and KCNE3, are discussed as essential possible pathogenetic factors [13-17]. However, in recent years, non-classic presentations of TPP have been increasingly published, particularly in cases with normal potassium levels [1,4,6,7,11,12].

Here, we report a case of a German adolescent of Vietnamese background with inconsequentially treated GD as a pre-existing condition, who presented with acute paralysis of the lower limbs but never showed significantly low serum potassium levels. To the best of our knowledge, this is only the second reported case of normokalemic TPP in the pediatric population [7].

## Case presentation

A 17-year-old adolescent boy of Vietnamese background, diagnosed with GD eight months ago based on positive antibodies and signs of GD on ultrasound, was initially prescribed thiamazol 5 mg once daily. However, after two months, he discontinued the medication. He reported experiencing muscle weakness during athletic activities over the past two years, but no diagnosis regarding paralysis was made.

The boy presented to the pediatric emergency unit due to acute sudden paralysis of the lower limbs (grade: 2-3/5), predominantly affecting the thighs and shanks bilaterally, without involvement of his feet. He also reported hypoesthesia in the lateral regions of his thighs, which began early in the morning. There were no meningeal signs or disturbance of bowel and bladder function. Before this onset, he experienced multiple episodes of vomiting and had a fever. On clinical examination, signs of an acute upper respiratory tract infection were noted (temperature up to 38.3°C, heart rate 105 bpm, reddened pharynx, and a florid left eardrum), along with an enlarged thyroid gland, brisk reflexes, and exophthalmos. Family history did not reveal any thyroid diseases or acute periodic paralysis. An urgent MRI of the spine and an ECG showed no significant abnormalities. Therefore, a diagnosis of proximally accentuated sensorimotor paralysis with an unclear focus was established.

Blood tests showed leukocytosis with neutrophilia in the complete blood count (CBC: leukocytes = 15.24/nl, reference = 4-11/nl and neutrophils = 13.6/nl, reference = 1.7-6.8/nl) and apparent hyperthyroidism (TSH < 0.01 mU/l, reference = 0.50-4.30 mU/l; fT3 = 21.84 ng/l, reference = 3.00-4.90 ng/l; and fT4 = 76.03 ng/l, reference = 9.50-16.40 ng/l). All electrolytes, including potassium (K), sodium (Na), magnesium (Mg), and phosphate (PO4), were within normal range. The electrolytes were measured in the hospital laboratory by the blood gas analyzer, which showed almost identical values (Table 1). Measures were taken to prevent pre-analytical errors as this is the hospital's standard. No deviation from these standards was reported. Shortly after hospitalization, the paralysis spontaneously resolved, leading to full recovery after approximately 12 hours. The patient was started on thiamazol 15 mg once daily.

**Table 1:** Serum levels of electrolytes and thyroid function test BGA: Blood gas analysis; ref: Reference; u. sup.: Under K supplementation; Day#: With respect to the day of presentation in the hospital; ↓: Low; ↑: High; ion.: Ionized Ca; K: Potassium; Na: Sodium; Mg: Magnesium; PO4: Phosphate; TSH: Thyroid-stimulating hormone; TPO: Thyroid peroxidase; TG-AB: Thyroglobulin antibody.

Labs	Day 1 (BGA)	Day 1	Day 2 (BGA)	Day 2	Day 2	Day 3 (BGA)	Day 3	Day 10	Day 24
K (mmol/l) ref: 3.5-5.1	4.3	4.8	3.3 (↓)	3.4 (↓)	4.1 (u. sup.)	4.1 (u. sup.)	4.3 (u. sup.)	4.4 (u. sup.)	3.7 (u. sup.)
Na (mmol/l) ref: 136-141	133 (↓)	-	140	140	140	140	139	143	141
Ca (mmol/l) ref: 2.10-2.55	1.22 (ion.)	-	1.31 (ion.)	2.3	-	1.33 (ion.)	-	2.57 (↑)	2.31
PO4 (mmol/l) ref: 0.84-1.45	1.37	1.37	-	1.52 (↑)	1.13	-	1.53 (↑)	1.16	1.19
Mg (mmol/l) ref: 0.70-0.91	0.68 (↓)	0.68 (↓)	-	0.71	0.65 (↓)	-	0.74	0.83	0.73
Cl (mmol/l) ref: 95-110	104	-	109	-	-	-	-	-	-
fT3 (ng/l) ref: 3.00-4.90	-	21.84 (↑)	-	-	-	-	-	-	-
T3 (μg/l) ref: 0.91-2.18	-	-	-	-	-	-	-	1.59	1.27
fT4 (ng/l) ref: 9.50-16.40	-	76.03 (↑)	-	-	-	-	-	20.11 (↑)	13.1
TSH (mU/l) ref: 0.50-4.30	-	<0.01 (↓)	-	-	-	-	-	<0.01 (↓)	<0.01 (↓)
TSH-R.-AB (U/l) ref: <1.50	-	9.89 (↑)	-	-	-	-	-	8.81 (↑)	7.14 (↑)
TPO-AB (kU/l) ref: <34.0	-	>600 (↑)	-	-	-	-	-	-	-
TG-AB (kU/l) ref: <64.0	-	>4000.0 (↑)	-	-	-	-	-	-	-

On the second night of hospitalization, the patient experienced another episode, characterized by weakness in both lower limbs without paralysis (grade: 4/5), with the feet spared. Additionally, he complained of mild weakness in his upper limbs and palpitations. However, his respiration remained unaffected, and ECG findings were normal. Blood drawn during this episode revealed low but nearly normal potassium levels (3.3 mmol/l↓; reference: 3.5-5.1 mmol/l), with normal values for Na, Mg, Ca, and slightly elevated PO4 (Table 1). By morning, the weakness had spontaneously resolved.

Based on this episode, the diagnosis of atypical, normokalemic TPP was made, and the patient was started on oral potassium supplements. Given the recent initiation of thiamazol, a non-selective ß-blocker was not administered immediately as it was expected to become effective shortly, and oral potassium supplementation was associated with fewer side effects.

Electrolytes were regularly monitored thereafter, remaining predominantly normal. The patient was discharged the following day with an unremarkable neurological examination, normal ECG, and stable electrolyte levels. No further episodes of weakness or paralysis were reported. Subsequent blood analysis showed normal electrolyte levels and normalized T3 and fT4 (Table 1). TSH remained barely detectable.

Genetic testing related to mutated ion channels (KCNJ2, KCNJ18, KCNE3, SCN4A, and CACNA1S) was conducted, but the results did not reveal any significant pathological findings.

## Discussion

Our case represents an atypical normokalemic onset of TPP, a rare complication of hyperthyroidism, in an adolescent patient, with only a few reported cases in the literature. The cardinal symptom of TPP is distinctive paralysis in the lower extremities with spontaneous regression [1-8,11-13,18-20].

Hyperthyroidism appears essential for the pathogenesis of TPP. However, some reports declared TPP as the initial manifestation of thyroid disease, without other signs of thyrotoxicosis [5]. Currently, low potassium levels are assumed to contribute to the pathogenesis of TPP [1-8,11-13,18-20].

Al-Zubeidi et al. reported cases from three American-Hispanic teenagers with TPP, of which one showed only mild hypokalemia (3,2 mmol/l; reference: 3,5-5,1 mmol/l) [4]. Jung et al. reported a 16-year-old Korean patient who was hypokalemic (2,7 mmol/l; reference: 3,5-5,1 mmol/l) in his first episode of paralysis but showed normal potassium levels (4,6 mmol/l; reference: 3,5-5,1 mmol/l) at consultation after a second episode [5]. Satam et al. indicated, in their case report of a 10-year-old girl, that late diagnosis of TPP in normakalemic events can have lethal consequences [7]. Normokalemia was also reported in some adult patients with episodes of TPP [6,11,12].

Chakrabarti described a case of a 27-year-old patient, who was initially normokalemic during his first onset of TPP, but his potassium level decreased constantly afterward until supplementation was started [6]. A similar process could have occurred in our patient. Although we did not measure the decline of the potassium level between the first and the second episodes of TPP, the potassium level clearly decreased in between until we started a supplementation.

Jung et al. reported that the potassium concentration in the specific concerned muscle cells is more crucial than the general intravascular potassium concentration. Between the attacks, the patient is often normokalemic and shows no residual neuromuscular symptoms [5]. Al-Zubeidi et al. and Jung et al. propagated that in the recovery stage, potassium levels can be even elevated (rebound hyperkalemia) or high normal because of a counter-regulation [4,5].

While hypokalemia is often associated with TPP, absolute potassium levels may not solely determine its pathogenesis. It is hypothesized that rapid intravascular potassium decline, rather than absolute low potassium levels, leads to symptoms. Our patient exhibited a rapid decline from initially 4.8 mmol/l in his first event to 3.3 mmol/l at the onset of the second episode. The significance of an initially high normal potassium range in the blood remains debatable.

It is important to note the lack of standardization in measuring potassium levels relative to the time point of testing, which may influence findings of hypo- and normokalemia described in various studies, leading to atypical normokalemic presentations of TPP. In contrast, hypophosphatemia or hypermagnesemia may also be relevant during TPP episodes [1]. However, significant evidence referring to this was not found in our case.

Our patient exhibited typical TPP symptoms and met several risk criteria but deviated from typical age and electrolyte alterations. Under potassium supplementation and antithyroid therapy, no new episodes occurred, supporting the hypothesis of an atypical TPP case.

To confirm the diagnosis and assess familial risk, we conducted a genetic analysis of ion channels associated with TPP using the next-generation sequencing. Several ion channel genes are discussed to play an essential role in TPP pathogenesis, such as KCNJ18, KCNJ2, KCNE3, SCN4A, and CACNA1S [4,13-17].

Kir 2.6, encoded by the KCNJ18 gene, represents a main pathologic factor in the common hypothesis of TPP pathophysiology [10]. Mutations in this gene may disrupt potassium efflux regulation, leading to paralysis. KCNJ2, KCNE3, SCN4A, and CACNA1S encode further membrane transport proteins for potassium, sodium, and calcium.

Our genetic analysis did not show pathogenic/likely pathogenic variants or variants of unclear significance, which is consistent with previous research [16]. Therefore, the significance of mutations in these receptor genes remains uncertain in our patient and requires further investigation to find the genetic etiology.

The pathogenesis of TPP remains incompletely understood, though dynamics of blood potassium levels seem to play a major role. Further research, including serial blood tests conducted near or before the onset of paralysis, is crucial to examine the exact pathogenesis of TPP and its relationship to potassium levels.

## Conclusions

In conclusion, TPP should be considered in the differential diagnoses of sudden acute sensory-motor paralysis in children and adolescents with known hyperthyroidism as it could lead to harmful complications. As no safe provocation tests, genetic analysis, or direct blood markers are known, thyroid function tests should be conducted in every unclear case. The diagnosis should rely on history and clinical elements with a high index of suspicion. Lastly, patient education about the possible complications of newly diagnosed hyperthyroidism should include information about the clinical picture and triggers of TPP as well as immediate measures that should be taken in the case of acute TPP.
